# Inhibitory effect of 405 nm laser light on bacterial biofilm in urethral stent

**DOI:** 10.1038/s41598-023-30280-0

**Published:** 2023-03-08

**Authors:** Luluil Maknuna, Van Nam Tran, Byeong-Il Lee, Hyun Wook Kang

**Affiliations:** 1 Industry 4.0 Convergence Bionics Engineering, Pukyong National University, Busan, Korea; 2 Marine-Integratedntegrated Biomedical Technology Center, The National Key Research Institutes in Universities, Pukyong National University, Busan, Korea; 3 Division of Smart Healthcare, College of Information Technology and Convergence, Pukyong National University, Busan, Korea; 4 Department of Biomedical Engineering, Pukyong National University, Busan, 48513 Republic of Korea

**Keywords:** Microbiology, Urology

## Abstract

The clinical use of urethral stents is usually complicated by various adverse effects, including dysuria, fever, and urinary tract infection (UTI). Biofilms (formed by bacteria, such as *Escherichia coli*, *Pseudomonas aeruginosa*, and *Staphylococcus aureus*) adhering to the stent cause UTIs in stented patients (approximately 11%). The undesirable consequences of antibiotics use include bacterial resistance, weight gain, and type 1 diabetes, which occur when antibiotics are used for a long time. We aimed to assess the efficacy of a new optical treatment with a 405 nm laser to inhibit bacterial growth in a urethral stent in vitro. The urethral stent was grown in *S. aureus* broth media for three days to induce biofilm formation under dynamic conditions. Various irradiation times with the 405 nm laser light were tested (5, 10, and 15 min). The efficacy of the optical treatment on biofilms was evaluated quantitatively and qualitatively. The production of reactive oxygen species helped eliminate the biofilm over the urethral stent after 405 nm irradiation. The inhibition rate corresponded to a 2.2 log reduction of colony-forming units/mL of bacteria after 0.3 W/cm^2^ of irradiation for 10 min. The treated stent showed a significant reduction in biofilm formation compared with the untreated stent, as demonstrated by SYTO 9 and propidium iodide staining. MTT assays using the CCD-986sk cell line revealed no toxicity after 10 min of irradiation. We conclude that optical treatment with 405 nm laser light inhibits bacterial growth in urethral stents with no or minimal toxicity.

## Introduction

Urethral stents (USs) are used to treat bladder outlet obstruction caused by various diseases. Urethral stricture disease, benign prostatic hyperplasia (BPH), and detrusor sphincter dyssynergia (DSD) are indications for US insertion in eligible patients^1^. Typically, US is constructed of a metal alloy (nitinol), polymeric materials, or biodegradable material that is robust enough to maintain urethral patency. Culha et al. have reported long-term US efficacy of 63% in patients with recurrent urethral strictures^2^. Therefore, the unsuccessful result of stent insertion, such as stent migration, encrustation, chronic urinary infection, urethral pain, and restenosis, are the complications of stent placement^3^. Riedl et al. discovered that all patients with chronic indwelling stents have stent colonization and bacteriuria. Temporary stents are also widely colonized, with a 69% occurrence rate, and 45% of patients are affected by bacteriuria; such microbial colonization causes UTI^4^. UTIs are caused by Gram-positive and negative bacteria, such as *Escherichia coli*, *Klebsiella pneumoniae*, *Proteus mirabilis*, *Enterococcus faecalis*, and *Staphylococcus aureus (S. aureus)*, and certain fungi^5^.

Urinary infection can occur when sterile US is implanted into the human body. Biofilm refers to an accumulation of bacteria and their extracellular byproducts that form an organized community on the surface^6^. Biofilm growth significantly impacts foreign objects or equipment implanted in the human body. A wide range of the foreign bodies, such as US, implantable device, sling, have been developed for urological applications over the past few decades^6^. Thus, on the surface of an implant, the biofilms are highly structured and comprise actively growing populations of bacteria, proteins, and extracellular polymers. The increased biofilm formation can result in bacterial resistance to antibiotics and chronic infections^7^.

Currently, antibiotics and stent surface coatings are used to prevent urinary infections^8^. Although most antibiotics are common, some antimicrobials have potential side effects if used for a long period of time^9^. The risks of using antibiotics include bacterial resistance, obesity, and diabetes^10^. The adverse effects of using antibiotics may include malabsorption resulting in a celiac-like disease, reduced medicine absorption, altered metabolism and absorption of vitamins, colonization by resistant organisms, and altered susceptibility to infections^11^. Laser application for bacterial inhibition via optical fibers is gaining attention as an alternative method to address these issues^12,13^.

A wide range of wavelengths can be applied (from 400 to 1000 nm), and among the wavelengths, blue light has shown effectiveness in reducing the viability of a variety of bacterial species, including methicillin-resistant *S. aureus* (MRSA), *Helicobacter pylori, Porphyromonas gingivalis*, and *Pseudomonas aeruginosa*, while most spectra at high irradiance are able to kill bacteria by photothermal effect even with low irradiance^14,15^. Another laser source, such as ultraviolet (UV) light, is also well recognized for bactericide. However, due to cytotoxic effects on mammalian tissue, the in vivo use of UV light is restricted^16^. Dai, et al. discovered a 57% loss of keratinocytes when researching UV light therapy for central line infections^14^. This cytotoxicity indicates that despite being an effective anti-microbial agent, the application of the UV light may not be the ideal choice for living tissue^17^. Thus, 405 nm laser light (blue light) has been widely used for bacterial disinfection^18,19^. Maclean et al. concurred that oxidative damage to the bacteria and photoexcitation of porphyrins at the 405-nm wavelength might generate the highest germicidal activity^20,21^. In addition, porphyrins and other photoactive substances can decontaminate bacteria by generating reactive oxygen species (ROS) as a result of the absorption of the 405-nm light. ROS is a class of free radicals comprising of singlet oxygen, superoxide anion, hydrogen peroxide, and hydroxyl radicals. During bacterial cytotoxicity, under the violet-blue light exposure (405 nm), endogenous photosensitizing molecules (porphyrins) get photo-excited and generate ROS. The sufficient production of ROS leads to cellular lysis via oxidative stress. In general, the production of ROS in bacteria involves two pathways during photodynamic therapy (PDT): Type 1 and Type 2. In Type 1, endogenous porphyrin reacts directly with the cellular components, including protein, lipids, nucleic acids, and other micro molecules, to generate superoxide anion and hydroxyl radical, which, in turn, leads to the production of other ROS molecules. In type 2, porphyrin binds with molecular oxygen to form singlet oxygen. This mechanism subsequently leads to oxidative damage to light-exposed bacterial cells and causes cellular death^21,22^. Other mechanisms, such as the disruption of bacterial membranes and DNA damage, have been reported to be the effects of blue light^23^.

Although antibiotics has been used for treating UTIs, the current medication is still associated with various side effects, such as obesity and bacterial resistance. The aim of the current study was to examine an alternative treatment for UTI by irradiating laser light on the bacterial biofilm grown in US via an optical device. It was hypothesized that using 405 nm laser light could inhibit bacterial biofilm as a phototherapy in stented patients to prevent UTIs. This in vitro study was designed as a first-stage investigation for the alternative treatment of UTIs. A 405 nm laser from a basket-integrated optical device was used to deliver light onto the inner surface of the US. For safety evaluations, MTT assays were used to evaluate the effect of 405 nm laser light as a phototherapeutic agent to inhibit bacterial biofilm formation in human cell lines. The proposed optical treatment can be a feasible alternative treatment for bacterial infection in stented patients.

## Results

### Bacterial culture in US

**Figure 1:**
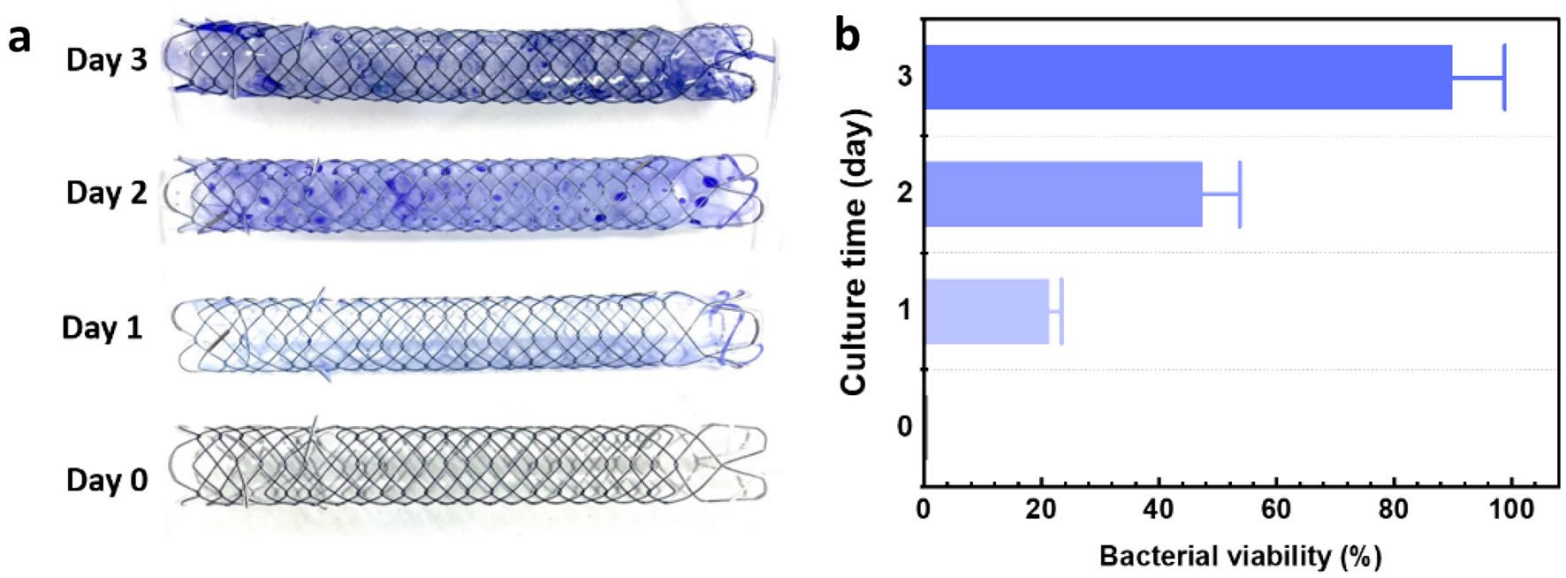
Growth of bacterial biofilm: (**a**) urethral stents stained with crystal violet at various time points after culturing and (**b**) bacterial viability versus culture time.

The bacterial culture condition in the US was analyzed by using the CV staining method to estimate the approximation of bacterial viability during the culturing time. Each day offers the different biofilm formations, based on Fig. 1a (0–3 day culturing). The CV staining worked as an intercalating dye, which is used for fast biofilm visualization, and the stianing allowed the quantification of cells, as shown in Fig. 1b. The estimation of biofilm formation on day 3 reached more than 80%, compared to that on day 0.

### Evaluation of effect 405 nm laser in human cell lines (CCD-986sk)

**Figure 2:**
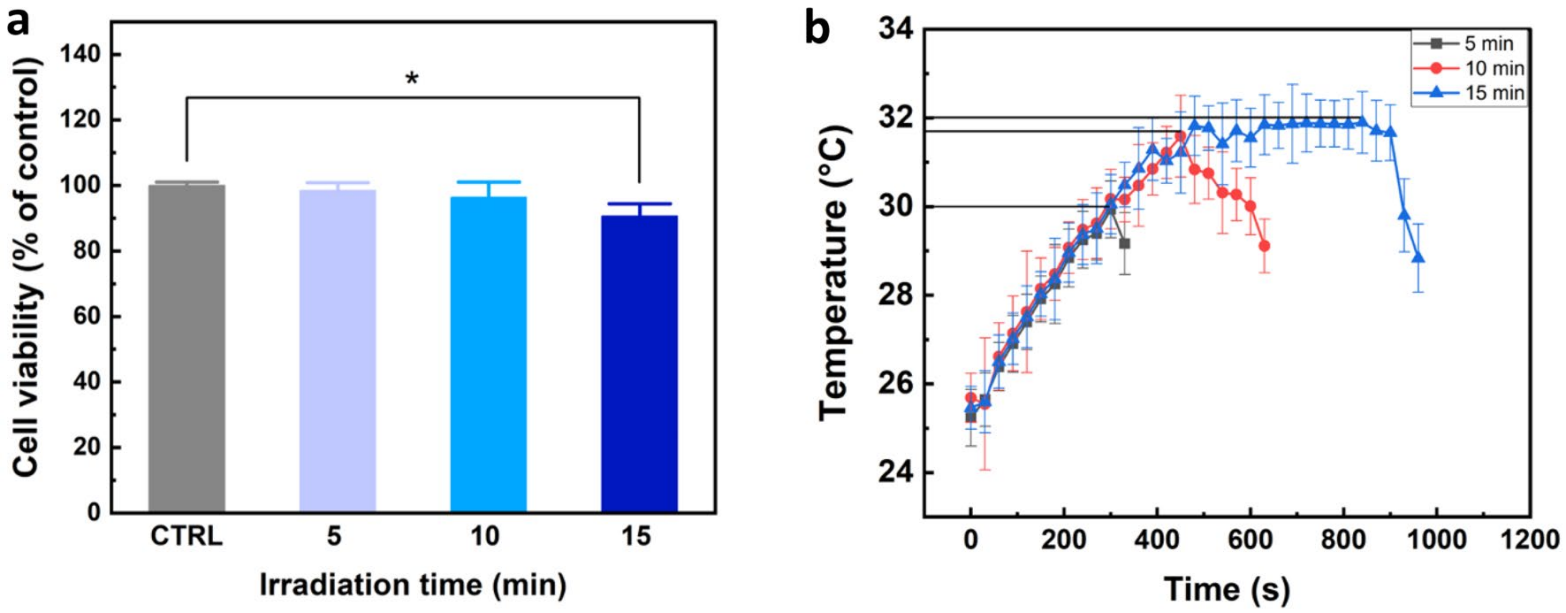
Response of normal cells (CCD-986sk) to 405 nm laser irradiation: (**a**) cell viabilities of various irradiation times (CTRL = control; (^*^p < 0.05 vs. CTRL; N = 5) and (**b**) temperature development during laser irradiation on surface of CCD-986sk suspension in DMEM media.

An MTT experiment was carried out to evaluate cell viability under both pre-and post-treatment settings to select an alternative method of removing the bacterial biofilm from US with 405 nm laser light. Treated cells showed the decreased cell viabilities of 2% (5 min), 4% (10 min), and 10% (15 min) after 24 h of incubation, in comparison to control (*p* < 0.05). The MTT assay results demonstrated that the 15 min laser irradiation caused toxic effect to the cells, leading to more than 10% cell reduction (Fig. 2a). Temperature development in Fig. 2b demonstrates that the maximum reached a temperature of 32 °C for 15-min irradiation.

### Evaluation of bacterial inhibition in US

**Figure 3:**
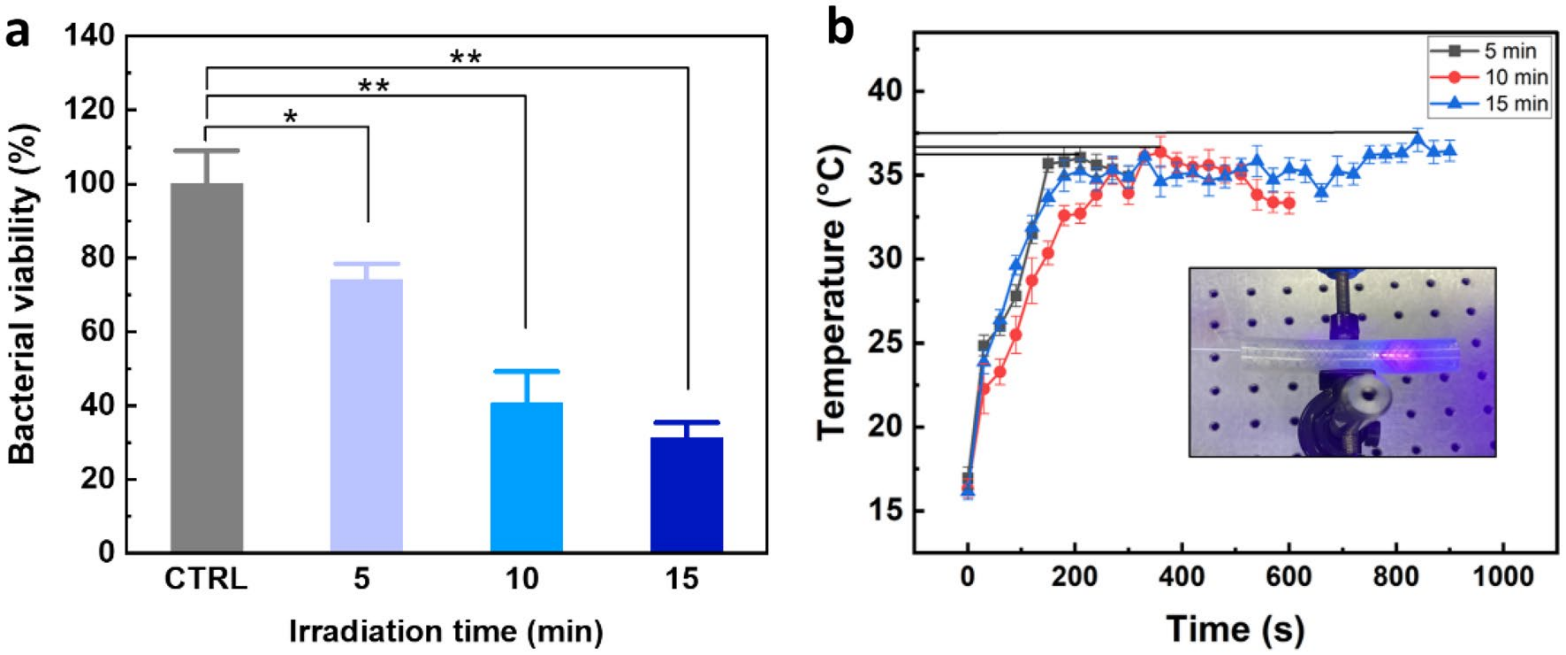
Inhibition of bacterial biofilm after 405 nm laser irradiation: (a) bacterial viability after exposure of 405 nm laser light at various irradiation times (CTRL = control; ^*^p < 0.05 and ^**^p < 0.001 vs. CTRL; N = 5) and (b) temperature development during laser irradiation on urethral stent surface.

CV staining was utilized to calculate the bacterial survival before and after laser treatment in order to evaluate the inhibitory impact of 405 nm laser light on *S.aureus*. As represented in Fig. 3a, the bacterial viability decreased by 26% in 5 min (*p* < 0.05), 59% in 10 min (*p* < 0.001), and 68% in 15 min (*p* < 0.001), respectively. The maximum temperature on the stent surface reached 37.5 °C (Fig. 3b).

**Figure 4:**
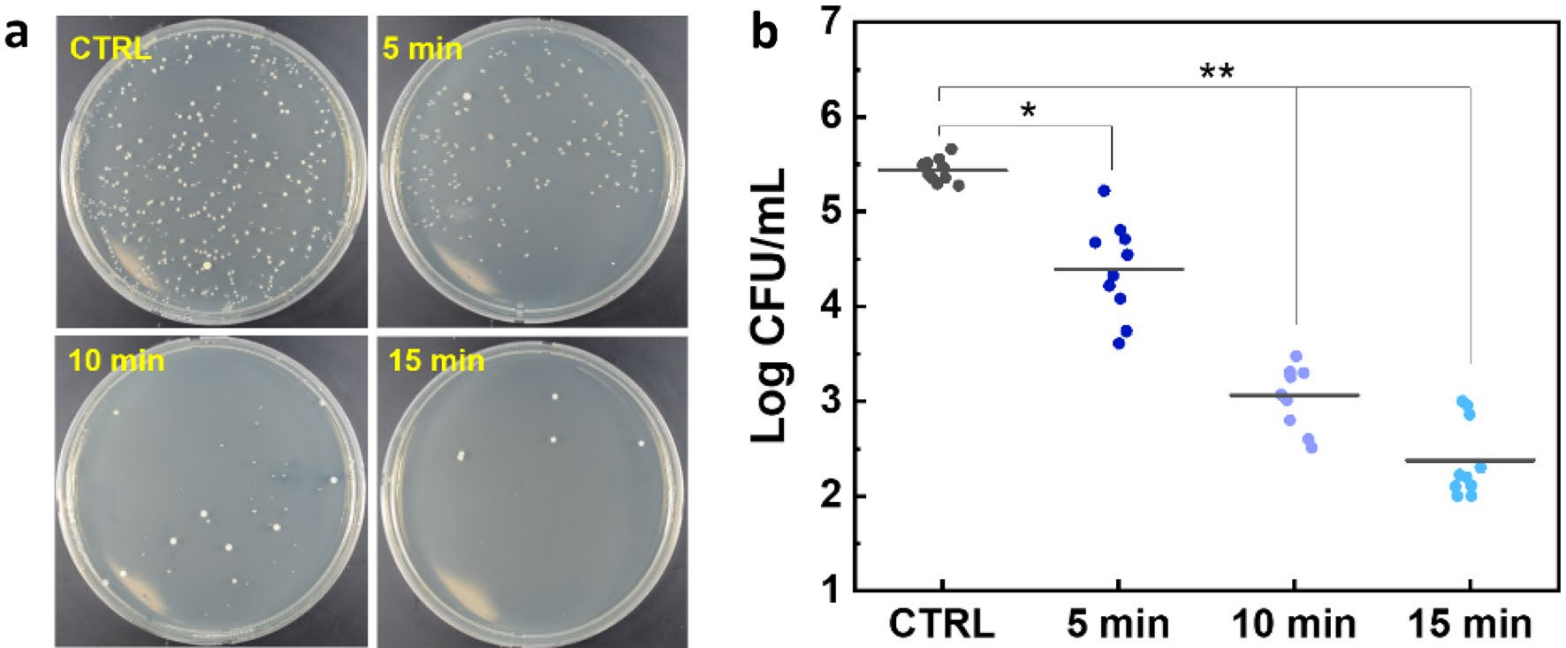
*S. aureus* bacterial inhibition after exposure of 405 nm laser at various irradiation times: (**a**) bacterial cells on agar plates (CTRL = control) and (**b**) quantification of bacterial removal in log CFU/mL (^*^p < 0.05 and ^**^p < 0.01 vs. CTRL; N = 10).

Figure 4a shows the determination of *S.aureus* in CFU after 405 nm irradiation, and each treatment condition depicts a different number of colonies (N = 10). The results showed that the total colony decreased after the irradiation and was represented in log CFU/mL (1.2 log, 2.2 log and 3.2 log reduction for 5, 10, and 15 min) (Fig. 4b). Although the maximum inhibition reached a 3.2 log reduction (*p* < 0.01) in 15 min irradiation (270 J/cm^2^), the human cell viability (CCD-986sk) (Fig. 2a) under the same condition also decreased by more than 10%. Thus, the 10 min laser irradiation could accomplish the minimum inhibition rate (2–3 log reduction) to prevent the biofilm formation in US with the safety treatment dose.

**Figure 5:**
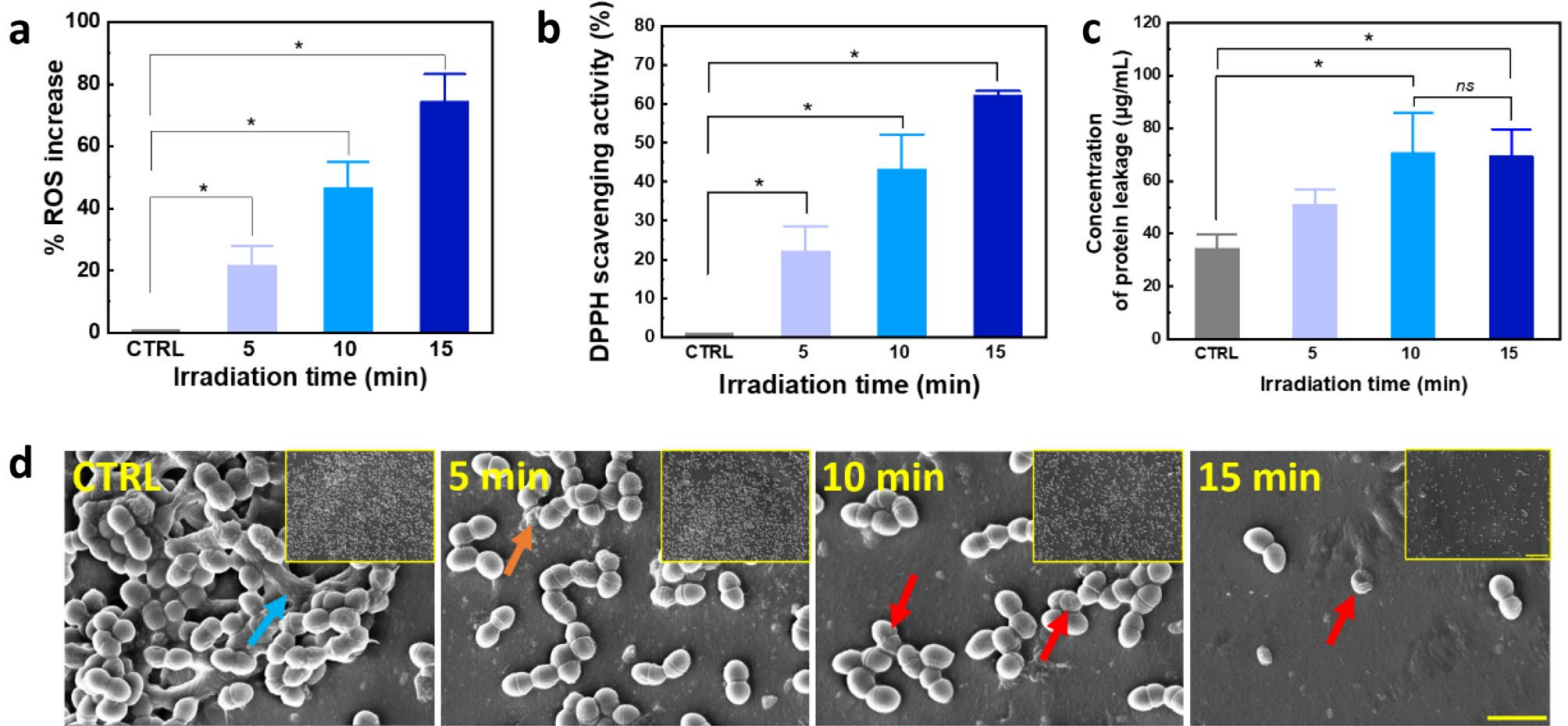
Optical inhibition effect of 405 nm laser light against *S. aureus* in urethral stent: (**a**) ROS generation during treatment, (**b**) DPPH scavenging activity, and (**c**) concentration of protein leakage using Lowry method (^*^p < 0.05 and ^**^p < 0.001 vs. CTRL; ns = not significant; N = 5), and (**d**) SEM images of *S. aureus* after laser treatment (blue, orange, and red arrow represent thick biofilm, bacteria released from biofilm, and bacterial death with morphological damage (scale bar = 2 µm; 20 kX and scale bar of inlet = 20 µm; 2 kX).

Figure 5a presents the levels of ROS generation determined by NBT staining, which was entered into the cell membrane. The result shows that the NBT could detect the ROS generation in all treated conditions. The results of ROS generation were 21.7, 46.7, and 74.4% (*p* < 0.05) for 5, 10, and 15 min, respectively. According to this result, longer irradiation of 405 nm laser could produce more ROS that affected cellular death. Figure 5b demonstates the result of the scavenging activity of antioxidants by using the DPPH assay. The results demonstrates that the free radicals after the ROS production increased, based on the irradiation time of 405 nm laser light. The treatment conditions (5, 10, and 15 min) reached 22% (*p* < 0.05), 43% (*p* < 0.001), and 62% (*p* < 0.001) of antioxidant activity compared to control, respectively. The control group (no laser treatment) showed the smallest antioxidant capacity whereas the treated groups yielded the increasing antioxidant capacities with the increasing light doses. The bacterial group that received the greater dose of 15 min revealed a statistically significant increase in the DPPH scavenging activity (*p* < 0.05) in comparison to the control. Figure 5c presents the concentration of protein leakage before and after treatment by using the Lowry method. The observation of the concentration of protein leakage in 5, 10, and 15 min reached 37, 71, and 69 µg /mL, respectively. The largest amount of protein leakage after the laser treatment was measured in 10 min of the laser irradiation and decreased in 15 min. The protein leakage activity reached a stationary state after 10 min of treatment because the protein concentration dropped in 15 min of irradiation, compared to 10 min laser irradiation (*p* = 0.92). These findings suggested that the 405 nm laser could enhance bacterial death by causing bacteria to undergo the increased oxidative stress and allowing the proteins to leak out of the damaged bacterial membrane.

**Figure 6:**
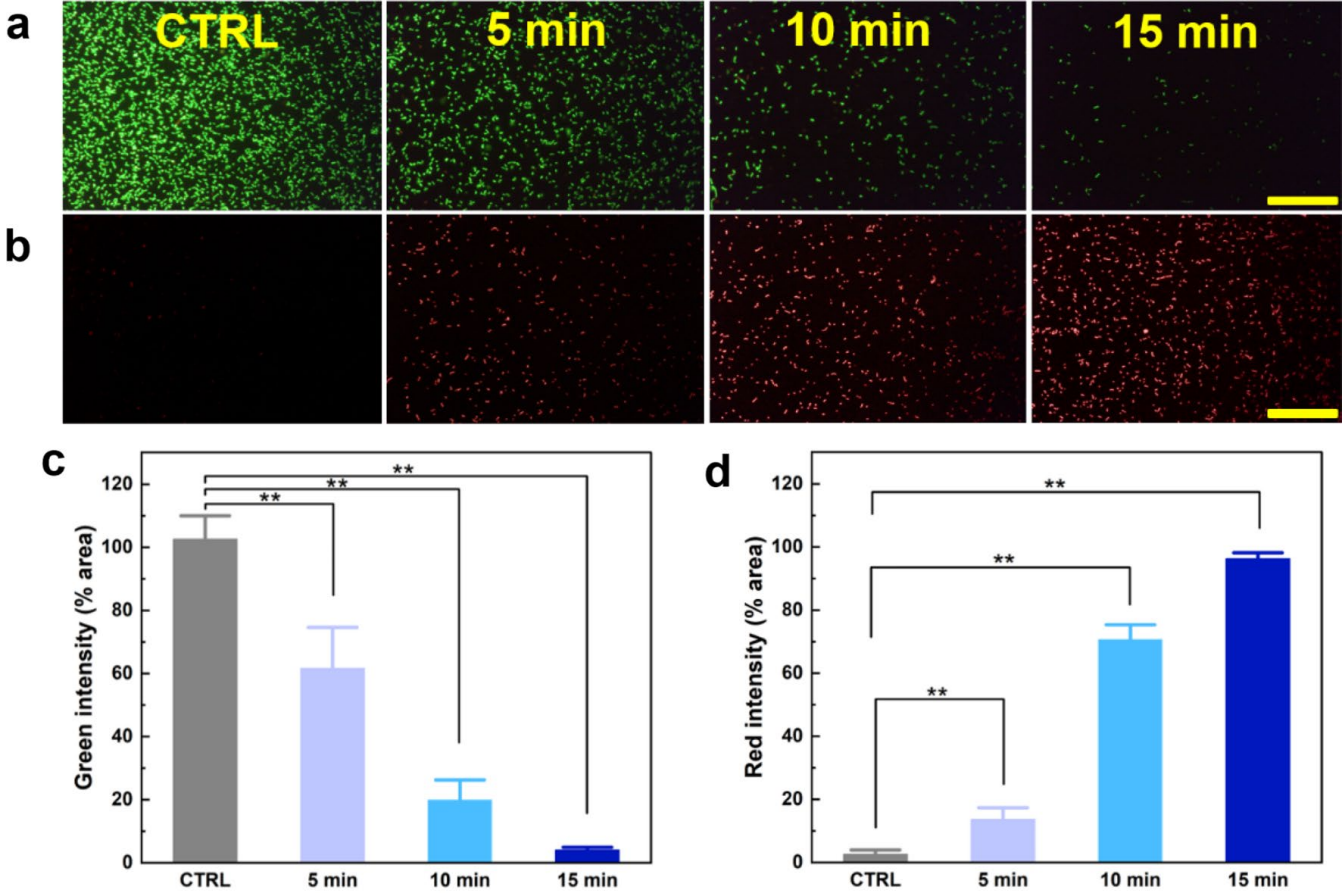
Analysis of bacterial membrane integrity after laser treatment: (**a**) live bacteria stained with SYTO 9, (**b**) dead bacteria stained with PI, (**c**) quantification of pixel intensity for live bacteria (green intensity), and (**d**) quantification of pixel intensity in dead bacteria (red intensity) (^**^p < 0.001 vs. CTRL; ns = not significant; N = 7; scale bar = 100 µm; 40X).

The membrane integrity loss of the bacterial biofilms exposed to four different treatment conditions are shown in SEM (Fig. 5d) and fluorescent images in Fig. 6 (CTRL, 5, 10, and 15 min). A significant proportion of bacterial colonies have developed into a thick biofilm architecture made of extracellular polymeric materials (blue arrow) in the untreated control (Fig. 5d (CTRL)). According to Fig. 5d (5 min), the 405 nm laser light destroyed the biofilm and released the biofilm into a single cell (orange arrow). The bacterial colonies in the biofilm deposited in the US showed the absence of intact biofilm morphology. In Fig. 5d (10 and 15 min), some bacteria were damaged (red arrow), indicating cell death, and the bacteria were removed from the US.

Fluorescent image assesment indicated that the implementation of 405 nm laser light significantly decreased the number of bacterial cells with few intact cell membranes remaining. The SYTO 9 marked the remaining intact membrane as live bacteria (green color), and the population after the treatment was lower than that of control (Fig. 6a). Propidium iodide (PI) demonstrated that the number of the bacteria after the treatment was higher than that of the control, which means more membrane damage represented in red color after the treatment (Fig. 6b). The quantification of live bacteria (Fig. 6c) represented in green intensity confirmed that the 405 nm laser light could inhibit the bacterial biofilm in US, which agrees well with the results of SEM images (62, 21, and 4% for 5, 10, and 15 min (*p* < 0.001), respectively). The quantification of dead bacteria validated that the 405 nm laser light could inhibit the bacteria that obtained higher red intensity after the 405 nm laser exposure (17, 68, and 98% for 5, 10, and 15 min , respectively) (Fig. 6d).

## Discussion

The current study attempted to understand the effect of 405 nm laser light against the bacterial biofilm-developed US. The laser treatment was used as an attempt to replace or assist the use of antibiotics to prevent the side effects. Another treatment to prevent the UTIs is stent removal, but along with that, trauma and other risks, such as major urological complications after stent removal and the urethral obstruction, still occur^24,25^. Thus, the alternative treatment, such as laser irradiation, can be another option to prevent UTIs.

405 nm laser light has an ability to inhibit bacterial biofilm formation in the US. The mechanism to inhibit the biofilm formation relies on ROS generation, which can affect membrane disintegration and cell death^26,27^. According to a prior work^28^, endogenous porphyrins in bacterial cells selectively absorbed 405 nm laser light and produced the intracellular ROS required to effectively kill the bacteria. Additionally, the presence of ROS can result in lipid peroxidation, protein and nucleic acid oxidation, and enzyme inhibition, all of which may destroy microorganisms^21^. Giuliano et al. reported that a typical patient under a urologic procedure with a positive urine culture has 10^3^ colonies^29^. The bacterial reduction from the present work confirmed that the inhibition using the 405 nm laser light could reduce more than 3 log reduction after 15 min of laser exposure with an irradiance of 0.3 W/cm^2^. Although the condition was insignificantly toxic to normal cells (Fig. 2), the safety dose should still be confirmed for this treatment prior to clinical translations. Bauer et al. reported the cytotoxic effects were found at 300 J/cm^2^ after 405 nm laser exposure^30^. An MTT assays performed after 15 min laser irradiation (270 J/cm^2^) also confirmed cytotoxic effects with more than 10% cell reduction. Therefore, the 10-min laser irradiation of 405 nm laser light could be the safe dose (180 J/cm^2^) with less than 10% cell reduction and 2.2 bacterial log reduction.

ROS generation after 405 nm laser treatment was examined by using NBT staining, which interacts with superoxide ions to produce an insoluble and stable intracellular purple/blue formazan precipitate^31^. The production of ROS is affected by the exposure time of the 405 nm laser to bacteria. The longer light exposure is applied to the bacteria, the more ROS can be detected in each condition. In addition, scavenging activity affected by ROS was carried out by DPPH assay, which yielded similar results to ROS generation after the laser treatment. The capacity of the 405 nm laser that interacts with bacteria to donate hydrogen to DPPH and convert it to DPPH-H is considered to be a free radical scavenging efficacy. This phenomenon resulted in a decrease in the absorbance value after the radical purple color (DPPH) was changed to yellow color (DPPH-H)^32^. Moreover, the effect of 405 nm laser light against the bacterial cells was confirmed by using the estimation of protein leakage by Lowry’s method. Interestingly, the concentration of protein leakage after the treatment increased, but for 15 min irradiation, the concentration of protein starting decreased and resulted in the lower amount of protein release possibly because of the disintegration of the protein molecules by the prolonged laser irradiation. The largest amount of the protein concentration occurred in 10 min with 71 µg/mL. Since the protein is a type of essential intracellular component^33^, protein leakage is considered to be an indication including both cytoplasmic leakage, damaged of lipid layer^34^ and cell membranes^35^.

The effect of 405 nm laser on morphological cells were dependent on the 405 nm laser irradiation time, resulting in biofilm removal and cell damages in 10 and 15 min. The structure changes from the perfect circle to shrunk circle, caused by the membrane disintegration which confirmed by SEM images of bacteria. One criterion for determining whether bacterial cells are alive or dead is thought to be cellular and membrane integrity. The live cells are supposed to have the intact and tight cell membranes that are impermeable to some stainings, whereas the dead cells are assumed to have disrupted and/or damaged membranes^36^. SYTO 9 staining bind to DNA and RNA and emit green flurescence while PI can only enter cells with the compromised membrane, which binds to DNA and RNA and emits the red fluorescence signals^37^. The membrane integrity loss after 10 and 15 min laser irradiation had a lower green pixel intensity and has a higher red pixel intensity compared to control. It was confirmed that the 405 nm laser caused the membrane disintegration of the bacteria and emited the red fluorescence signals from the dead bacteria after the treatment.

For clinical applications, the proposed method is expected to be used for patients with UTI by inserting a basket-integrated optical device into the urethra and positiong it on the US. The diffusing optical fiber situated in the urethral channel can irradiate 405-nm laser light onto the surface of the in-dwelling US to prevent or minimize the formation of bacterial biofilm on the US with photo-inhibitory effects. Further works should thus test an in vivo model to explore clinical doses and to adapt the optimal dose to a urethral channel and peripheral tissues. The minimum inhibition rate to remove 10^3^ colonies could be different under the real-time condition.Thus, it is necessary to carry out experiments under the conditions that can mimic the urethral channel. In addition to make it one of the treatments for UTI in stented patients, we need to clarify and monitor the photo-inhibitory effect against urological diseases, such as urethral obstruction and urethral stricture. Lastly, a pilot study should be performed to identify and optimize the clinical treatment conditions, such as laser power, irradiation time, and number and period of treatment, as well as to validate the efficacy and safety for clinical translations.

In conclusion, the present work demonstrated the effectiveness of a 405 nm laser light for bacterial inhibition in US with 2–3 log bacterial reduction and the safety margin. The ROS generation after 405 nm laser exposure to the biofilm contributed to protein leakage, membrane disintegration, and affected cell death. Further studies will examine the simultaneous bacterial inhibition using the multi-bacterial in vivo to mitigate the risk of UTI in stented patients by reducing the presence of bacterial resistance caused by the prolonged use of antibiotics.

## Materials and methods

### Bacterial biofilm formation

**Figure 7:**
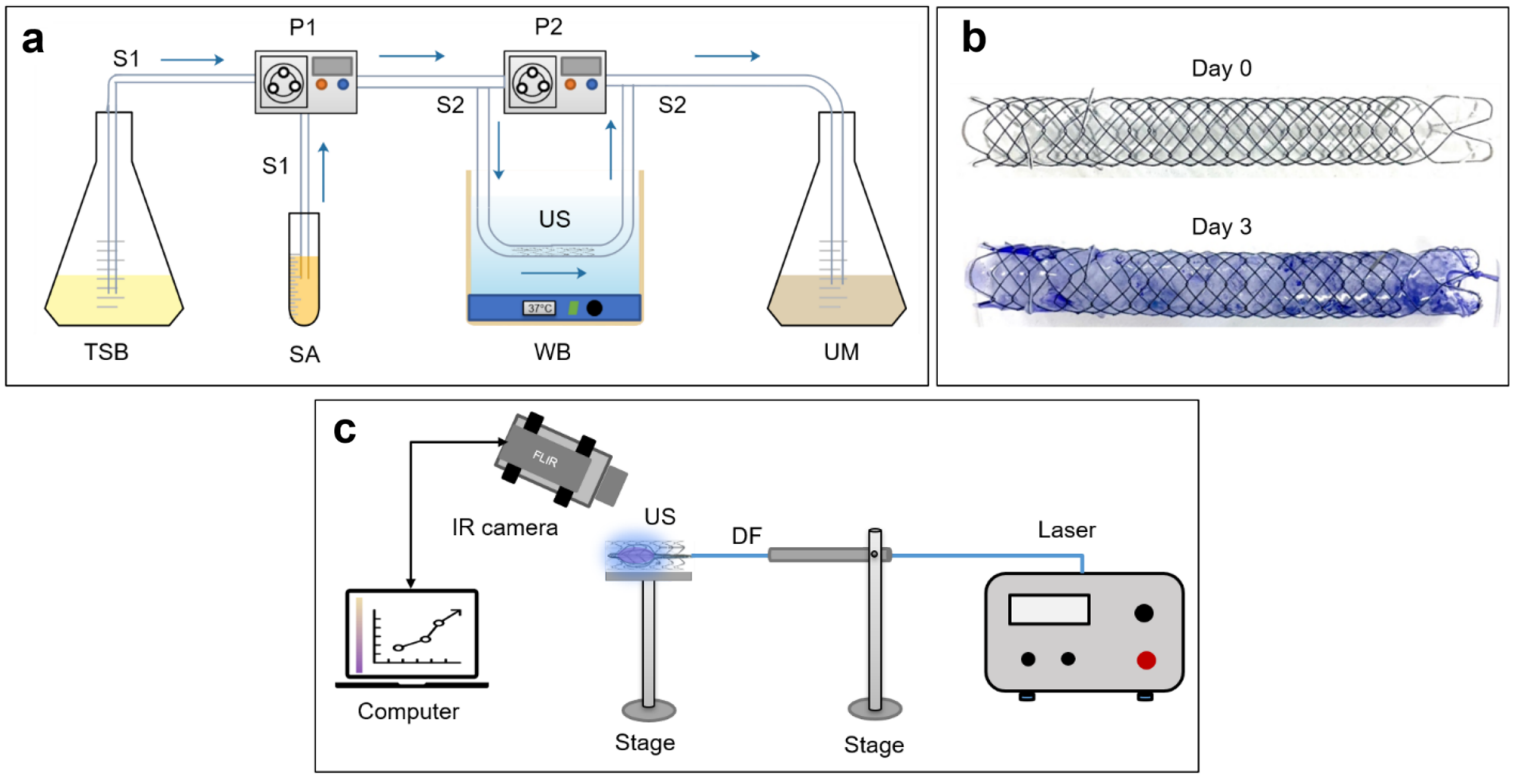
Schematic illustrations of bacteria preparation: (**a**) bacterial culturing in urethral stent under dynamic condition, (**b**) examples of urethral stent before (day 0) and after culturing, and (**c**) 405 nm laser treatment set up. (day 3; stained with crystal violet; TSB = tryptic soy broth media; SA = *S. aureus* suspension; WB = water bath; US = urethral stent; UM = used media;P1, P2 = pump, S1 = small silicone, S2 = large silicone, and DF = diffusing fiber).

Sterile USs were purchased from UVENTA™ Urethral stent; TAEWOONG Medical, South Korea. The US was self-expandable, with 80 mm in length and 10 mm in diameter,and consisting of an implantable full-coverage metallic structure made of nitinol wire and covered with a thin layer of silicone (not biodegradable). *S. aureus* (KCTC 1916) from bacterial stock culture (− 80 °C) were placed in an agar plate and inoculated for 24 h. Then, the bacteria were subcultured in tryptic soy broth (TSB) (50 mL) and incubated for 24 h in an incubator at 37 °C. To determine the number of bacterial colonies, the McFarland method^38^ was used to obtain 10^8^ colonies in 10 mL. Initially, fresh medium was pumped (P1 = 1.5 mL/min) in a small silicone tube (inner diameter = 4 mm; outer diameter = 5 mm; Sungjin, South Korea) with bacterial suspension injection (10 mL). Then, the second pump (P2 = 50 mL/min) was turned on when P1 drained the media throughout the silicone tube, and was used to drain the larger silicone media (inner diameter = 10 mm; outer diameter = 12 mm; Sungjin, South Korea) for three days. A US was placed in a larger silicone tube, and the silicone was placed in a water bath at 37 °C. Subsequently, the media that was used flowed into another bottle. Fresh media was pumped every 4 h with P1 to keep the bacteria from growing and forming a uniform biofilm in the US. It should be noted that the current process was focused on the development of the bacterial biofilm in the US in vitro, which hardly mimics clinical conditions. Figure 7A shows the setup. After being cultured for three days, the US was stained with crystal violet (CV) to determine whether the biofilm was formed during the culturing days, as shown in Fig. 7b.

### Experimental setup

The basket-integrated optical device used in this study was adapted by modifying the basket shape, diameter (10 mm), and the characterization of the material that was used for the basket device from previous work^39^. The basket, which was integrated with an optical diffuser, aims to maintain the position of the diffusing fiber in the center when placed in the US, such that the spread of light can be uniform in all directions. Blue light (405 nm; CNI laser, Changchun, P.R. China) was used as the light source with an irradiance of 0.3 W/cm^2^. The treatment conditions were varied, depending on the irradiation times of 5, 10, and 15 min. Control was a non-treated condition. The corresponding fluences were 90, 180, and 270 J/cm^2^. For the treatment area, we cut the stent into 10 mm in length and exposed the 405 nm laser light inside the stent. An experimental setup was developed by placing the cultured US in a standing holder, and a basket-integrated device was then positioned in the same direction and inserted into the US at the center. An infrared camera (FLIR A325, 320 × 240 pixels, resolution = 25 μm, spectral range = 7.5–13 μm; FLIR, Wilsonville, Oregon) was placed 30 cm above the US surface and controlled with a personal computer to monitor temperature development during treatment (Fig. 7c).

### MTT assay

The 3-(4,5-dimethylthiazol-2-yl)-2,5-diphenyltetrazolium bromide (MTT; Sigma- Aldrich, St. Louis, MO, USA) assay was used to evaluate safety and toxicity of the 405 nm laser light. The CCD-986sk cell line (procured from the Korean Cell Line Bank) was used as the normal cell line in all treatment conditions. CCD-986sk cells were thawed in a water bath at 37 °C, placed in a 15 mL conical tube, 3 mL of Dulbecco’s modified Eagle’s medium (DMEM, Corning, NY, USA) was added, and the suspension was centrifuged at 1000 × *g* for 5 min. The supernatant was removed, and the cells were placed in a culture dish with 10 mL DMEM for 48 h. Cell subculture was performed every two or three days to obtain a stable cell line for the MTT assay. The stable cell lines were counted, seeded in 24-wells plates, and incubated for 24 h to allow the cells to grow well before treatment. The cells were treated according to the treatment conditions applied to the US (irradiation times: 5, 10, and 15 min) with an irradiance of 0.3 W/cm^2^. Treated cells were incubated for 24 h. After the incubation, the medium was removed, and MTT solution was added and incubated for 3 h in the dark at 37 °C. The MTT solution was removed, replaced with dimethyl sulfoxide (DMSO), and incubated for 15 min. The absorbance was determined spectrophotometrically at 570 nm using a microplate reader (Multiskan GO, Thermo Fisher Scientific, Waltham, Massachusetts, USA; N = 10 per condition).

### Bacterial viability

After treatment, CV staining was used to estimate bacterial viability by washing the US with distilled water and incubating for 20 min in CV solution. Subsequently, a solubilization solution was added (95% ethanol with 5% distilled water) and sonicated for 10 min. The bacterial solutions were placed in a 96-well plate, and its absorbance at 570 nm was measured using a microplate reader (Multiskan GO, Thermo Fisher Scientific, Waltham, Massachusetts, USA; N = 10 per condition). In addition, bacterial viability was calculated in terms of colony-forming units (CFU). Ten millimeters of US were washed with 1 mL distilled water and placed in a 15 mL conical tube. US was sonicated for 10 min and vortexed for 5 min. The bacterial suspension was then diluted tenfold, and 100 μL of the suspension was spread on a tryptic soy agar (TSA) plate and incubated for 24 h at 37 °C. After incubation, agar plates were analyzed using OpenCFU^40^ to calculate the number of bacterial colonies, which is represented by the log number of the bacterial viability on the 10 mm US surface (CFU/mL, N = 10 per condition).

### Evaluation of oxidative stress

To investigate the dominant mechanism of the proposed treatment, both the control and treated US were placed in a 15 mL conical tube with 1 mL distilled water, sonicated for 20 min, and vortexed for 5 min to detach the bacteria in the US. A 1 mg solution of nitro blue tetrazolium (NBT) was added to the tube and incubated for 30 min in the dark. After 0.1 M HCl was added to the solution to inhibit bacterial interaction with NBT, all tubes were centrifuged at 12,000 × *g* for 5 min. To release intercellular ROS, the pellets were first treated with 800 mL of saline and 400 mL of dimethyl sulfoxide (DMSO). To estimate ROS generation, 200 μL of each sample was placed in a 96-well plate and measured at 575 nm (N = 5 per condition). The collected ROS generation was normalized by the absorbance of control samples to exclude any experimental errors caused by the sonication procedure. The following equation was used to determine the level of intercellular ROS amplification (Ri):

$$ R_{i} = \left[ {\frac{{\left( {At - Ac} \right)}}{Ac} \times 100} \right] $$

where Ac is the absorbance of the control and At is the absorbance of the treated sample.

### DPPH scavenging activity

1,1-Diphenyl-2- picrylhydrazyl (DPPH) is a stable free radical used to evaluate the general radical-scavenging capabilities of various antioxidants after ROS generation. DPPH assays were performed by adding the DPPH solution (40 µg/mL methanol) to the control and treated cells and incubating for 30 min in the dark. A UV spectrophotometer was used to detect the absorbance at 517 nm, and the experiment was repeated five times. A log dosage inhibition curve was used to obtain the standard’s IC_50_ value^41^, which is the concentration of the standard required to inhibit 50% of the DPPH free radical. The reaction mixture’s lower absorbance suggested the increase free radical activity. The following equation was used to calculate the percentage of the DPPH scavenging (D_s_) effect.

$$ D_{s} = \left[ {\frac{{\left( {Ac - At} \right)}}{Ac} \times 100} \right] $$

where Ac is the absorbance of the control, and At is the absorbance of the treated sample.

### Protein leakage

The method described by Lowry et al. (1951) was used to assess protein released from *S. aureus* cells (10^6^ CFU/mL) before and after treatment with 405 nm laser light for 24 h. Absorbance was measured spectrophotometrically at 660 nm. The amount of released protein was determined by extrapolating an equation from the calibration curve using bovine serum albumin (BSA), as previously described^42^.

### Microscopic evaluation of biofilm formation

To evaluate biofilm formation architecture, control and treated US were washed several times with distilled water and fixed using 2.5% glutaraldehyde with pH 7.5 for 4 h at room temperature (26 °C). After incubation, the US was washed with 100% ethanol and left to dry at room temperature. The control and treated US were cut into small pieces (5 mm) and covered with gold–palladium for SEM analysis.

A LIVE/DEAD BacLight Bacterial Viability kit (Bio Probes, Eugene, OR, USA) was used to evaluate live and dead bacteria based on the membrane integrity of the control and treated stents. The bacterial suspension from the control and treated US was centrifuged at 1000 rpm for 5 min to collect the bacterial biofilm attached to the stent. SYTO 9 and propidium iodide (PI) stains were then added at a 1:1 concentration and incubated for 15 min. After incubation, 1 μL suspension was added to a glass slide and covered with a coverslip under a fluorescence microscope. Live and dead bacteria are represented in green and red, respectively. A quantitative analysis of the fluorescence images was performed by using the total number of green and red pixel intensities and calculating the RGB values with Image J (National Institute of Health, Bethesda, MD, USA).

### Statistical analysis

Data are represented as the mean and standard deviation under four conditions: one as a control and three other treatment conditions (irradiation times: 5, 10, and 15 min). Each condition (control and treatment) was repeated five times (N = 5). CFU analysis was performed in 10 replicates to ensure appropriate data. The Mann–Whitney *U* test was used to evaluate all conditions by comparing the control and type conditions. SPSS (SPSS, Chicago, USA) was used for statistical analysis, and *p* < 0.05 was considered statistically significant.

### Ethics approval

This article does not contain any studies with human participants performed by any of the authors.

## Data Availability

All relevant data used to support the findings of this study are included within the article. Additional information and data are available from the author (LM; maknuna@pukyong.ac.kr) upon reasonable request.
